# The Relative Age Effects in Educational Development: A Systematic Review

**DOI:** 10.3390/ijerph18178966

**Published:** 2021-08-26

**Authors:** Alar Urruticoechea, Andrés Oliveri, Elena Vernazza, Marta Giménez-Dasí, Rosario Martínez-Arias, Javier Martín-Babarro

**Affiliations:** 1 Facultad de Psicología, Universidad Católica del Uruguay, Montevideo 11600, Uruguay; 2 Dirección General de Planeamiento, Universidad de la República, Montevideo 11200, Uruguay; andres.oliveri@udelar.edu.uy; 3 Instituto de Estadística, Facultad de Ciencias Económicas y de Administración, Universidad de la República, Montevideo 11200, Uruguay; evernazza@iesta.edu.uy; 4 Sección Departamental de Investigación y Psicología en Educación, Facultad de Psicología, Universidad Complutense de Madrid, 28040 Madrid, Spain; magdasi@ucm.es (M.G.-D.); jbabarro@psi.ucm.es (J.M.-B.); 5 Departamento de Psicobiología y Metodología en Ciencias del Comportamiento, Facultad de Psicología, Universidad Complutense de Madrid, 28040 Madrid, Spain; rmnez.arias@psi.ucm.es

**Keywords:** age differences, developmental differences, education, educational process, systematic review

## Abstract

There is a large number of variables, studied in the literature, that affect the integral development of students in the educational stage, but few research analyze the effects that relative age can have on development. The aim of this study is to review and summarize the results obtained, on this subject, in recent research. The methodology used has followed the PRISMA declaration. The final sample is composed by 21 articles, which use data from 24 countries and 32 assessments. The main conclusions indicate that relatively younger children in same class groups: (a) obtain significantly lower mean scores in cognitive and motor tests, (b) have a higher repetition rate, and (c) have a less capacity of socialization. Finally, it should be noted that considering the results obtained by the research on relative age effect on child development, some authors propose to adapt educational practices to minimize these effects.

## 1. Introduction

The study of the relative age effect (RAE) on the development of cognitive, motor, and emotional skills in schoolchildren has gained momentum in recent decades worldwide, driven by the implementation and modification of public policies aimed at improving the teaching and learning processes inherent in any education system [1,2,3].

Relative age is defined as the difference in age between two or more subjects within a date range. In education, it will be the difference in age between schoolchildren in the same class [4,5,6]. In the absence of a single general rule on the criterion of the date of entry into the education system across countries, it is necessary to define what is meant by relative age on a case-by-case basis. There are two ways of calculating relative age: by calendar year and by arbitrary cut-off. In the former, we find, for example, the case of the Spanish education system, which segments each school year by those born in a certain year. Thus, schoolchildren born in January will be the “relatively older” and those born in December will be the “relatively younger”. The second has to do with the cut-off date imposed by the country. For example, Japan proposes to enrol children born before 1 April, so that schoolchildren born in April will be the “relatively older” and those born in March will be the “relatively younger”. In either scenario there will be a difference of almost 12 months between the “relatively younger” and the “relatively older” [7]. There is no theoretical framework of the RAE in educational development in the literature, but there is an approach to a theoretical framework from a sport perspective [8]. This proposes a model based on restrictions (constraints-based) that follow the essential interactional triangular structure of Newells framework [9]. Within this framework, individual, task and environmental limitations are considered, (a) individual limitations: inside these limitations it is considered the date of birth, the difference between chronological and biological age [10], the genre [11]. (b) Depending on the characteristics of the task and the ability required the relatively older may have advantage over the relatively younger and vice versa, for example in physical task, the relatively older have better performance in force task, while the relatively younger have better performance in artistic task [12]. This is also observed in reading abilities the RAE attenuates before (9 years) than in maths (11 years) [13]. (c) Environmental limitations: politics and a grouping system (where the relative age is located), level of maturity and influence of the family [14,15]. Although this theoretical framework focuses on sports, could be considered inside educational development, since it is focused on cognitive development (individual limitations), the motor (task limitations), and socio emotional (environmental limitations).

Although most studies have demonstrated the influence of relative age on academic performance [16,17,18], relative age is still not a variable that is taken into account when organizing school groups or analyzing academic outcomes [19]. The academic achievement can be defined as the level of knowledge, skills, and abilities that a student acquires throughout his or her educational life [20]. It can be assessed by the results obtained in tests that measure academic achievement, for example: international tests (PISA), national tests, school exams or repetition rates. Socio-emotional skills are defined as the abilities to recognise and regulate one’s own emotions, as well as to identify and understand the emotions of others in contexts of social interaction [21,22]. Social competence refers to the demonstration of self-efficacy in social transactions [22]. This self-efficacy requires the previously mentioned emotional competences and the capacity for empathy. These skills, together with other dispositional and biological aspects, can foster favorable or unfavorable development for school children [23]. In this aspect, adequate socio-emotional development is considered to be that in which the schoolchild has an appropriate management of emotions, both at an intrapersonal and interpersonal level, always depending on the context in which he finds himself. Interpersonal emotions are those that allow them to relate to their environment, among which are empathy and compassion. Intrapersonal emotions are those that the subject puts into consideration with himself, which allow an adequate reaction to contextual stimuli, among which, in the academic field, motivation can be highlighted. Finally, behavioral skills are those manifested by the schoolchild in the face of socio-emotional difficulties that arise throughout life, so it can be considered that social skills and behavioral skills have a direct relationship [24]. Finally, motricity is the faculty or power of movement by the body or a body part [25]. On the one hand, there are movements that use large muscles, which make it possible to run and sit up straight, among other things. These movements are referred to as gross motor skills. On the other hand, there are the more specific movements, which require more refined skills, such as tying the buttons on a shirt and grasping a pencil, among other actions. These movements make up what is known as fine motor skills. Given that in the evolution of motor development, gross and then fine motor skills develop first, and that the acquisition of these skills varies according to the relative age of the schoolchild, public policies on entry, grouping, grading, and selection in education may disadvantage relatively younger schoolchildren, for example, in physical education performance [11]. It should also be taken into account that physical tests in the academic setting may be disadvantageous for these schoolchildren [26]. Based on the existence of the RAE on child development, it is essential to observe its prevalence and long-term consequences in the educational sphere, taking into account these factors in order to determine the importance of relative age in the integral development of the subject. In view of the above, this systematic review will aim to examine the effects caused by relative age and its prevalence, with special emphasis on the educational setting.

## 2. Materials and Methods

### 2.1. Search Strategies

To achieve the objective of the systematic review, we followed the guidelines set out in the PRISMA statement [27]. First, the objects of study, school entry age, and RAE, and the objective of the review were defined. We also established the search period, which runs from September 2019 to January 2020. The search was conducted using the following databases: Web of Science (WoS), PsycInfo, and SCOPUS. Taking into account that each search engine has different search options and with the intention of equating the searches, in WoS the option “Subject” was used, in PsycInfo the option “any field” and in SCOPUS the option “Article title, Abstract, keywords”. The terms used for the searches were “school entry age” and “relative age effects” using the Boolean connector “and”.

### 2.2. Inclusion Criteria

The criteria for inclusion of articles for the review were the following: (a) articles published in refereed and indexed scientific journals, (b) articles published between 1999 and 2019, (c) articles that compare the relative age in the sample they worked with, (d) articles whose research is based in formal education contexts (preschool, primary, secondary, or university), (e) articles that are not a systematic review, and, finally, (f) articles that do not consider populations with any learning difficulties or developmental disorders.

### 2.3. Data Collection

**Figure 1:**
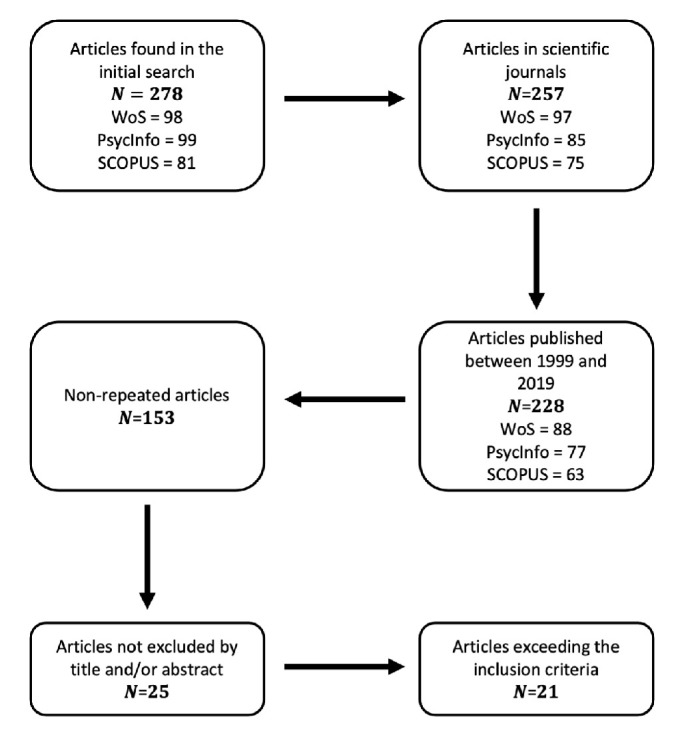
Data collection process.

A total of 278 articles were obtained, distributed by database as follows: 98 articles in WoS, 99 articles in PsycInfo, and 81 articles in SCOPUS. Once the scientific article filter was applied, 257 articles remained. To these we applied the publication date filter, articles published after 1999 and before 2019, leaving 228 articles. In addition, eliminating repeated records left a sample of 153 unique articles. Subsequently, a reading of the titles and abstracts of the articles was carried out, eliminating all those that did not compare relative age, thus obtaining a total of 25 articles. Finally, the papers were read and 4 articles, where the sample did not belong to a formal education group, were eliminated, leaving a total of 21 articles (see Figure 1).

### 2.4. Coded Data

Following the methodology of similar systematic reviews (Falla and Ortega-Ruiz, 2019), from the total of 21 articles obtained for the analysis of the results, the following categories were considered: (a) year of publication of the article, (b) authors of the article, (c) country of origin of the sample, (d) sample size, (e) age of the individuals in the sample, (f) instruments used to obtain results, and (g) main results of the research.

## 3. Results

Taking into account the 21 articles, the following results were obtained (see Table 1 and Table 2).

### 3.1. Year and Country of Study

**Figure 2:**
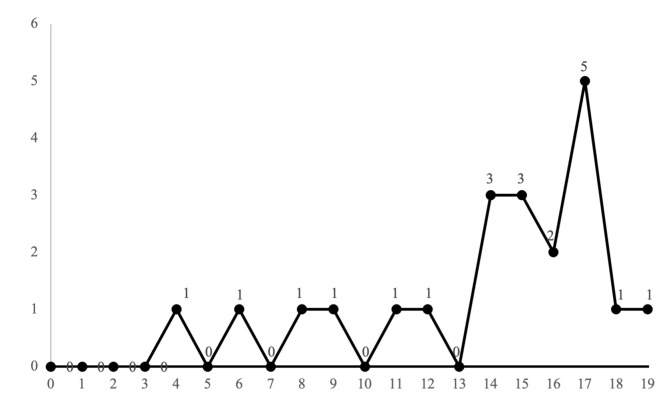
Number of articles published per year (1999–2019).

In the last twenty years, publications on the RAE on the educational environment have been scarce (21), with 2017 standing out as the year with the most publications, with 5 articles (see Figure 2).

**Figure 3:**
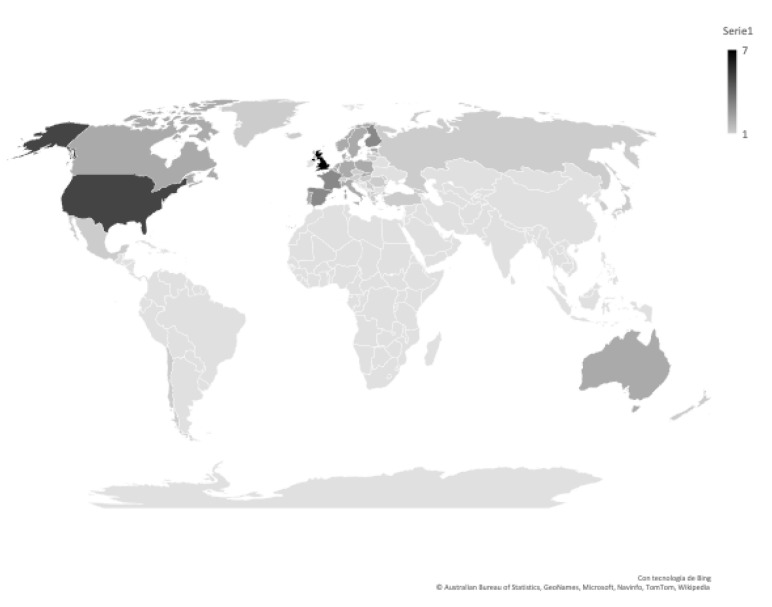
Articles published per countries.

The total number of articles is concentrated in 4 continents (Europe, America, Oceania, and Asia), 47.62% were carried out with European samples involving the following countries (order: highest to lowest occurrence): England, Spain, Finland, Germany, Belgium, Denmark, Slovakia, Estonia, France, Holland, Italy, Luxembourg, Norway, Poland, Portugal, Sweden, Albania, Austria, Azerbaijan, Bulgaria, Cyprus, Slovenia, Georgia, Greece, Greenland, Croatia, Hungary, Ireland, Iceland, Latvia, Lithuania, Macedonia, Malta, Moldova, Czech Republic, Romania, Russia, Serbia, Switzerland, Turkey, and Ukraine; 38.10% with American samples involving the following countries (order: highest to lowest occurrence): United States, Canada, Chile, and Mexico; 9.52% with Asian sample involving the following countries (order: highest to lowest occurrence): Japan and Korea; and finally, 4.76% with Oceanic sample, involving the following countries (order: highest to lowest occurrence): Australia and New Zealand (see Figure 3).

### 3.2. Sample Size and Study Target

**Figure 4:**
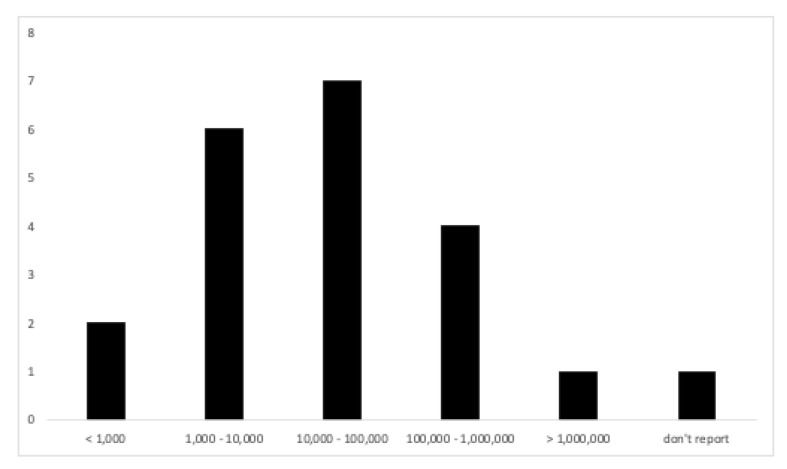
Number of published articles by sample size.

Of the total research, 9.52% used samples of less than one thousand participants, 28.57% used samples between one thousand and ten thousand participants, 33.33% used samples between ten thousand and one hundred thousand participants, 19.06% used samples between one hundred thousand and one million participants and, finally, 4.76% used samples of more than one million participants (4.76% do not report sample size) (see Figure 4).

### 3.3. Characteristics of the Educational Stage Studied

It is possible to divide the 21 research studies into two subgroups, those that focus only on one educational stage (66.66%) and those that include more than one stage for the study (33.34%). In the first case, of the total number of articles in the review, 9.52% focus only on the pre-school stage, 28.57% only on the primary stage, 23.81% only on the secondary stage, and 4.76% only on the university stage. In the second case, 4.76% study in pre-school, primary, and secondary, 19.06% study in primary and secondary, 4.76% study in primary, secondary, and university, and, finally, 4.76% study in secondary and university.

### 3.4. Instruments Used in the Studies

A total of 32 measurement instruments characterised as: (a) cognitive, (b) motor, and (c) socio-emotional were used in the 21 review articles. Cognitive assessments were used in 76.19% of the articles, with a total of 22 instruments. In 14.29% of the items, social-emotional assessments were carried out, with a total of 7 instruments. Finally, in 9.52% of the items motor assessments were conducted, with a total of 3 instruments.

## 4. Main Results of the Studies

### 4.1. Relative Age Effects on Academic Performance

The schoolchildren who delay their enrolment in a school year score significantly higher than schoolchildren enrolled in their corresponding cohort [28]. In the direction of this research, it can be asserted that relatively older schoolchildren in a primary school class (6–12 years) score significantly higher on academic achievement tests than their relatively younger peers [13,29,30]. This difference in scores could be the trigger for relatively younger schoolchildren to have the highest repetition rate [31,32], and there are differences in school completion rates in favor of relatively older schoolchildren [4,33]. It is worth noting that the RAE may be significantly larger when accompanied by risk variables, including economic, socio-demographic and family support variables. Thus, the RAE are larger at lower socio-economic levels [17,33,34], because the RAE are offset by the support of parents at higher socio-economic levels [35], so that schoolchildren at lower and relatively lower socio-economic levels will have lower test scores on academic achievement tests than relatively older schoolchildren. Taking gender into account, relatively younger male schoolchildren are more likely to score significantly lower than female schoolchildren of the same relative age [31,33]. If the coexisting variable with relative age is a person’s migration status in the country (demographic variable), immigrant or non-immigrant, it can be seen that relatively younger and immigrant schoolchildren obtain significantly lower mean scores than non-immigrant schoolchildren of the same relative age [6]. Finally, an adequate family climate of support and involvement in the education of relatively younger schoolchildren causes the RAE on academic performance to be significantly smaller than those of schoolchildren of the same relative age but with an inadequate family climate [6,28].

### 4.2. Relative Age Effects on Socio-Emotional and Behavioral Development

Taking relative age into account, it was found that, on the one hand, relatively older schoolchildren in a class have lower motivational levels than relatively younger ones [32]. On the other hand, relatively younger schoolchildren have lower levels of socio-emotional adjustment [36] and lower self-esteem [37] than relatively older ones. Moreover, maladaptive behavior is considered a risk factor for repetition and these behaviors occur to a greater extent in relatively younger schoolchildren [38], causing the repetition rate to be higher in this population compared to relatively older schoolchildren. Finally, in terms of interpersonal relationships, it is worth noting that while relatively younger students have fewer friends and meet with them less frequently, they communicate electronically more frequently than relatively older schoolchildren [16].

### 4.3. Relative Age Effects on Motor Development

The results presented by Roberts and Fairclough [3] show a slight difference in the mean scores obtained by 11–14 year-old schoolchildren in favor of the relatively older ones in each age range. The results of Prieto-Ayuso and Martínez-Gorroño [39] confirm the existence of these differences, asserting that relative age influences the basic physical abilities of male schoolchildren, but not those of female schoolchildren. They also state that while out-of-school physical activity influences the results obtained by schoolchildren and out-of-school sports activity does not, the difference lies in the objective of that activity: while physical activity aims to improve health in the long term and may not be regulated by strict rules (skating, cycling, playing with skittles, rescue-type games, etc.), sports activity aims to improve technique for an established sport, with strict rules and guided by a coach, monitor, or teacher (basketball, dance, tennis, etc.).

### 4.4. Prevalence of Relative Age Effects

The prevalence of RAE varies depending on the area of development being studied. In longitudinal research on achievement in general, differences that appear at the beginning of primary school are found to be compensated for as they progress through primary school [13] or when they enter secondary school, because relatively younger pupils have higher levels of concentration [40]. At age 12, there are still differences in favor of relatively older pupils; these differences disappear by age 15 [2]. Measuring academic performance only in the areas of mathematics and reading, the RAE found for 7-year-olds diminish at age 8 and disappear or even reverse by age 14 [6]. In terms of motor development, Roberts and Fairclough [3] report that the RAE disappear over the years, with students’ scores on physical education tests becoming equal during secondary education. Finally, addressing the potential long-term academic and economic repercussions of the effects of inadequate socio-emotional and behavioral development, Solli [35] claims that relatively younger males are less likely to be in college at age 25, as well as have lower levels of earnings, than their relatively older peers. Furthermore, Peña [5] states that differences in relative age have repercussions in adulthood, showing a tendency for relatively older people to have a higher level of education and greater savings capacity. Finally, it is noted that these results coincide with Sánchez Puerta et al. [41], who state that socio-emotional competencies are the best predictor of aspects such as the level of academic training, job opportunities, salary, health, satisfaction in social relations or psychological well-being in adult life.

## 5. Discussion

From this systematic review it can be concluded that, in the academic domain, there are significant differences in favor of relatively older schoolchildren. These differences are measurable in cognitive, motor, and socio-emotional performance. Although the RAE diminish at older ages, their long-term (adulthood) impact may be important in terms of further education, income and family savings capacity. The reasons why RAE disappear over the years have not been studied in depth, but repetition and dropout, motivated by low self-perception and self-esteem of relatively younger schoolchildren, could be causal variables, because only relatively younger schoolchildren who are better adapted to the demands of school tests will remain in the education system. It is worth mentioning that, as studies on early development indicate, all the variables that affect the infant during gestation and the first three years of life have enormous relevance for later development. During these years, the basic brain architecture that will impact the development of cognitive, emotional, social and linguistic skills of the child and the adult is configured [42,43]. In this sense, it seems reasonable to think that prematurity at birth will also influence development [44]. So much so that Perricone et al. [45] show that children born prematurely and without any disability have lower results in metacognition. If we add to this disadvantage the RAE, the disadvantages become more noticeable in the early stages of children’s development, which is another risk factor for school failure due to the mismatch between gestational and developmental age [46].

Finally, this systematic review has some limitations. First, a meta-analysis could not be performed due to the heterogeneity in the methodology. Furthermore, studied variables were different in each study (i.e, age, educational level, area of development studied, consequences, etc.), this prevented making any inference about the RAE to the population. Lastly, is the lack of research with a qualitative approach. This approach would allow not only to quantify the magnitude of the RAE but also to know the perception that students may have about these effects, the consequences and the possible solutions they see.

## 6. Conclusions

### 6.1. Implications for Educational Practice

Considering the need to act on the RAE in order to provide schoolchildren with the tools to achieve adequate cognitive, motor, and socio-emotional development, giving them greater confidence and understanding of the environment around them [47], different alternatives emerge:

- Change the grouping system at the time of entry to pre-school education. Make the allocation of classes more flexible, taking into account the differences in children’s development, depending on their relative age. Grouping by semester, facilitating mobility between the two groupings [48];
- Making tests more flexible, in two ways: first, by assessing children when they are exactly the same relative age, first the relatively older and then the relatively younger. Second, by standardizing test scores by relative age, comparing individual children with the scores of their peers [49];
- Promote late entry to relatively younger children [50];
- Implement educational strategies to avoid diminishing the self-esteem of students with low academic performance due to relative age [51].

These proposals could help mitigate repetition, dropout, and school failure associated with the RAE. It should be borne in mind, on the one hand, that these alternatives for grouping or specific activities to mitigate the RAE do not consist of generating two groups: one of “advanced” and another of “not advanced”, but to try to meet the demands and needs of similar groups that have homogeneous intra-group developmental rhythms. On the other hand, any educational change must be culturally accompanied in order to be understood and accepted by society.

### 6.2. Future Steps

Finally, it should be noted that there could be numerous alternatives to address the RAE on development in order to try to provide equal opportunities for all children. What seems clear is that to achieve this there is a need to adapt the school to the developmental characteristics of the children and not vice versa. It is, therefore, suggested that future research should be carried out:

- To study longitudinally the cognitive (performance), motor and socio-emotional development of schoolchildren from the beginning of their schooling until the disappearance of the RAE;
- To observe the level of influence of repetition and dropping out of school on the disappearance of the RAE;
- To analyze the differences of relative age in the scores obtained by age group, those born in the first semester on the one hand and those born in the second semester on the other;
- To examine the influence of teaching methodologies on RAE;
- Study the RAE from a qualitative perspective to know the experiences from the point of view of the different actors involved in the student development process.

## Figures and Tables

**Table 1:** Scientific articles taken into account in the systematic review (part 1).

Year	Author	Country	Sample	Age	Educational Stage	Instrument	Result
2004	Thompson et al.	CAN	1129	6;8, 9;11, and 12;14	Primary	Culture Free Self Esteem Inventory (CFSEI)	- Relatively younger students have lower self-esteem compared to relatively older students.
2006	Lawlor et al.	GBR	12,150	7, 9, and 11	Primary	Moray House Picture Intelligence Test (MHTPic), Schonell and Adams Essential Intelligence Test and Moray House Test	- Relatively younger students score significantly worse than relatively older ones at age 7. In reading literacy, the relative age differences were attenuated at age 9. In mathematics, the relative age differences attenuated at age 11.
2008	Abel et al.	USA	1375	<22	tertiary education	academic cognitive assessment	- Relatively older students apply more to medical school than relatively younger students. There is no difference in the percentage of acceptances between relatively older and relatively younger students.
2009	Martin	AUS	36,684	12;18	High school	Motivation and Engagement Scale–High School (MES-HS)	- Relatively older students are less motivated than relatively younger students. Relatively older students do not have a higher academic advantage than relatively younger students. Relatively older students have a higher repetition and dropout rate. Repetition effects increased these differences.
2011	Kawaguchi	JPN	14,727	9;10 and 13;14	Primary	The Trends in International Mathematics and Science Study (TIMSS)	- Relatively older students have higher academic scores than relatively younger students. The persistence of the effects indicates the importance of relative age in academic achievement.
2012	Roberts and Fairclough	GBR	582	11;18	High school	Academic physical assessment, Test of Gross Motor Development (TGMD-2)	- Relatively older students score higher than relatively younger students. These differences are small and may be due to decreasing relative age effects with increasing grade level.
2014	Aliprantis	USA	22,000	5	Preschool	Early Childhood Longitudinal Study (ECLS-K)	- These differences are small and may be due to decreasing relative age effects with increasing grade level.
2014	Crawford et al.	GBR	4668	7	Primary	Avon Longitudinal Study of Parents and Children (ALSPAC), Millennium Cohort Study (MCS), WISC, The national achievement tests (KS1)	- Relatively older students score higher than relatively younger students in cognitive and non-cognitive skills.
2014	Nam	KOR	6000	8, 14, 17, and 18	Primary, High school, and Vocational	Korean Education and Employment Panel (KEEP)	- Relative age effects are present up to the end of primary school. In secondary school, there are differences in favor of relatively younger students in the ability to concentrate and the ability to avoid distractions. Thus relatively younger students compensate for the difference in prior academic performance.
2015	Huang	USA	7441	5	Preschool	Early Childhood Longitudinal Study (ECLS-K)	- Relative age alone is not a sufficient factor to consider a student suitable or unsuitable for a high ability programme. Taking into account demographic variables such as gender, race and socio-economic status, relatively older students are more likely to be eligible.
2015	Navarro et al.	CHL	15,234	13	Primary	National System of Quality Assessment in Education Survey (SIMCE)	- Relatively older students have higher SIMCE scores. Socio-economic status and type of school have a greater impact on performance than relative age.
2015	Pehkonen et al.	FIN	3596	3, 6, 9, 12, 15, and 18	Preschool, Primary, High school	academic cognitive assessment	- Relatively younger students score lower than relatively older students at the end of sixth grade. By the end of ninth grade the differences, due to relative age effects, lose their significance. There are no significant differences in the length of time spent in formal education between students of different relative ages.
2016	Hemelt and Rosen	USA	1,194,856	10, 14, 15, and 18	Primary, High school, and tertiary education	Student-level administrative data	- Relatively younger students have a higher completion rate of secondary education than relatively older students. Relatively younger students have lower academic achievement scores than relatively older students. Relatively younger female students enrol three times more in college than relatively younger males. Relatively younger students enrol more in short courses (2 years) than relatively older students.

**Table 2:** Scientific articles taken into account in the systematic review (part 2).

Year	Author	Country	Sample	Age	Educational Stage	Instrument	Result
2016	Thoren et al.	DEU	80,946	7, 8, and 13	Primary	academic cognitive assessment	- Relatively older students score higher in reading and mathematics in second grade. The differences are still present in third grade, but are less strong. The differences disappear and even reverse in eighth grade. Relative age effects do not vary between students with and without immigrant ancestors.
2017	Dhuey et al.	USA	291,129	6;15	Primary and High school	academic cognitive assessment	- Relatively older children score higher on academic tests than relatively younger children. Relatively younger children have a higher repetition rate than relatively older children. Families from lower socio-economic backgrounds enrol their children earlier than those from higher socio-economic backgrounds.
2017	Diris	AUS, BEL, CAN, DNK, EST, FIN, FRA, ITA, LUX, NLD, NZL, POL, PRT, EVK, ESP, and SWE.	344,551	15	High school	PISA	- Relatively younger students score lower than relatively older students at the start of primary school. These effects are reduced in later years because relatively younger students learn faster than relatively older students in the first years of primary school. Because of these results it is not appropriate to decide whether a student repeats or not on the basis of his or her relative age. The negative impact of repetition in primary education outweighs the benefits. Late entry to school may have positive effects for the lower percentiles of the achievement distribution (especially girls).
2017	Peña	MEX	323,481	8;17	Primary and High school	Prueba Nacional de Logros Académicos en Centros Escolares (ENLACE)	- Relatively older students have an advantage in the tests compared to relatively younger students. If the assessment were completed asynchronously by relative age, relatively younger students would score higher than relatively older students. In adults, those of relatively older age will have more savings capacity and better educational attainment.
2017	Prieto-Ayuso and Martínez-Gorroño	SPA	174	12;16	High school	academic physical assessment	- There are no statistically significant differences between the mean physical test scores of students born in the first and second semester of the year.
2017	Solli	NOR	NA	13, 19, 25, and 30	High school and tertiary education	academic cognitive assessment	- Relatively older students have higher scores than relatively younger students. At lower socio-economic levels the relative age effects are stronger than at higher levels. Relatively younger males are less likely to complete Secondary Education than relatively older males. Relatively younger males are less likely to enrol in university at age 25. Relatively younger people, especially boys, have significantly lower earnings at age 30 than relatively older people.
2018	Dicks and Lancee	FRA	11,903	15;16	High school	PISA	- Relatively younger students with migrant parents are 10% more likely to repeat a grade in primary school. The effect sizes between these two variables (relative age and parental background) is larger than that between relative age and gender.
2019	Fumarco and Baert	ALB, AUT, AZE, BEL, GBR, CYP, CZE, DEU, DNK, EST, FIN, FRA, GBR, GEO, GRC, GRL, HUN, ISL, ITA, LVA, LTU, LUX, MLT, MDA, MKD, NLD, NOR, POL, PRT, ROU, RUS, SRB, SVK, SVN, ESP, SWE, CHE, TUR, and UK	423,575	10;16	Primary and High school	Health Behavior in School-Aged Children (HBSC)	- Relatively younger students communicate electronically more frequently than relatively older students. Relatively younger students have fewer friends and meet less frequently.

## Abbreviations

The following abbreviations are used in this manuscript:

RAE	Relative age effects
PISA	Programme for international student assessment
WoS	Web of Science
